# PiB Fails to Map Amyloid Deposits in Cerebral Cortex of Aged Dogs with Canine Cognitive Dysfunction

**DOI:** 10.3389/fnagi.2013.00099

**Published:** 2013-12-30

**Authors:** Rikke Fast, Anders Rodell, Albert Gjedde, Kim Mouridsen, Aage K. Alstrup, Carsten R. Bjarkam, Mark J. West, Mette Berendt, Arne Møller

**Affiliations:** ^1^Department of Clinical Veterinary and Animal Sciences, Faculty of Health and Medical Sciences, University of Copenhagen, Frederiksberg, Denmark; ^2^Centre of Functionally Integrative Neuroscience (CFIN), Aarhus University, Aarhus, Denmark; ^3^Department of Nuclear Medicine and PET Center, Aarhus University Hospital, Aarhus, Denmark; ^4^Department of Neuroscience and Pharmacology, Faculty of Health and Medical Sciences, University of Copenhagen, Copenhagen, Denmark; ^5^Department of Biomedicine, Faculty of Health, University of Aarhus, Aarhus, Denmark; ^6^Department of Neurosurgery, Aarhus University Hospital, Aarhus, Denmark

**Keywords:** canine cognitive dysfunction, Alzheimer’s disease, Pittsburgh compound B, beta-amyloid, dog, 6E10 immunohistochemistry

## Abstract

Dogs with Canine Cognitive Dysfunction (CCD) accumulate amyloid beta (Aβ) in the brain. As the cognitive decline and neuropathology of these old dogs share features with Alzheimer’s disease (AD), the relation between Aβ and cognitive decline in animal models of cognitive decline is of interest to the understanding of AD. However, the sensitivity of the biomarker Pittsburgh Compound B (PiB) to the presence of Aβ in humans and in other mammalian species is in doubt. To test the sensitivity and assess the distribution of Aβ in dog brain, we mapped the brains of dogs with signs of CCD (*n* = 16) and a control group (*n* = 4) of healthy dogs with radioactively labeled PiB ([^11^C]PiB). Structural magnetic resonance imaging brain scans were obtained from each dog. Tracer washout analysis yielded parametric maps of PiB retention in brain. In the CCD group, dogs had significant retention of [^11^C]PiB in the cerebellum, compared to the cerebral cortex. Retention in the cerebellum is at variance with evidence from brains of humans with AD. To confirm the lack of sensitivity, we stained two dog brains with the immunohistochemical marker 6E10, which is sensitive to the presence of both Aβ and Aβ precursor protein (AβPP). The 6E10 stain revealed intracellular material positive for Aβ or AβPP, or both, in Purkinje cells. The brains of the two groups of dogs did not have significantly different patterns of [^11^C]PiB binding, suggesting that the material detected with 6E10 is AβPP rather than Aβ. As the comparison with the histological images revealed no correlation between the [^11^C]PiB and Aβ and AβPP deposits in post-mortem brain, the marked intracellular staining implies intracellular involvement of amyloid processing in the dog brain. We conclude that PET maps of [^11^C]PiB retention in brain of dogs with CCD fundamentally differ from the images obtained in most humans with AD.

## Introduction

A prominent explanation of the etiology of Alzheimer’s disease (AD) is the amyloid cascade hypothesis (Hardy and Selkoe, 2002; Jack et al., 2010). According to this hypothesis, Aβ has a primary role in the neuropathological changes associated with AD. The deposition of Aβ is considered an early event in the pathogenesis of AD, implying that suitable biomarkers of Aβ load would detect early evidence of disease presence (McKhann et al., 2011).

Humans and dogs have coexisted in mutually beneficial partnerships for at least 100,000 years (Vila et al., 1997). Dogs develop signs of behavioral disorder that correlate with neuropathological findings, and aged dogs acquire behavioral deficits of spatial awareness, social interaction, sleeping pattern, house training, and memory and learning. This combination of behavioral symptoms is known as Canine Cognitive Dysfunction (CCD) (Satou et al., 1997; Rofina et al., 2006; Osella et al., 2007; Yu et al., 2011). The decline of cognitive functions correlates with an increase of Aβ deposits in the cerebral cortex of the dog (Colle et al., 2000; Pugliese et al., 2006a; Rofina et al., 2006). The Aβ deposits found in dog brains are primarily of the diffuse type and are of similar amino acid sequence to those observed in humans (Head et al., 1998; Sarasa et al., 2010). Dogs with CCD are therefore of special interest as an animal model of the early events of AD, when Aβ deposits mainly are of the diffuse type (Pugliese et al., 2006b; Rofina et al., 2006).

A modified form of the amyloid-binding histological dye thioflavin-T ([^11^C]PiB) made non-invasive PET imaging of amyloid deposits possible. Prior to this, visualization of Aβ deposits was only possible using histological post-mortem material (Klunk et al., 2004). Although new potential tracers are becoming available [^11^C]PiB is the most intensively evaluated marker of Aβ in human studies with PET (Klunk et al., 2004; Price et al., 2005; Mintun et al., 2006; Lockhart et al., 2007; Ikonomovic et al., 2008; Gulyas et al., 2012; Gjedde et al., 2013; Rodell et al., 2013). Compared to cognitively intact control subjects, AD patients exhibit greater [^11^C]PiB retention in areas known to contain substantial accumulations of Aβ deposits, including the frontal and parietal cortices, whereas brain areas relatively unaffected by Aβ pathology, such as the cerebellum, show little or no [^11^C]PiB retention (Klunk et al., 2004; Price et al., 2005; Ikonomovic et al., 2008).

The specific binding properties of [^11^C]PiB are still controversial, as is the correlation with the post-mortem histology of tissue taken from disease models established in animals (Klunk et al., 2005a; Toyama et al., 2005; Bacskai et al., 2007; Rosen et al., 2011). The tracer is known to pass the blood-brain barrier with comparative ease, which renders the uptake sensitive to blood flow differences, as well as amyloid load (Blomquist et al., 2008; Gjedde et al., 2013). Also, as a dye, Pittsburgh compound B (PiB) has varying affinities for different tissue components and for different multimeric assemblies of Aβ and Aβ precursor protein (AβPP). Thus, PiB binding to specific subtypes of amyloid is at variance with, and often much lower in mice and non-human primates, than in humans, despite substantial Aβ deposits in these species (Klunk et al., 2005b; Rosen et al., 2008; Manook et al., 2012). According to Rosen et al. (2010), it is possible that PiB recognizes a specific site in multimeric Aβ that is peculiar to most humans with AD, but may be unavailable in some humans and in other mammalian species.

It is not yet known whether tracer [^11^C]PiB detects Aβ deposits in dogs. To test if the tracer actually detects Aβ deposits in a cohort of dogs with a clinical diagnosis of CCD, in comparison with unaffected control dogs, and thus effectively reveals the known distribution of Aβ in this species, we obtained [^11^C]PiB PET images from the brains of animals in these two groups.

## Materials and Methods

According to the experimental design, PET images were obtained from dog brains using an HRRT CPS Innovations tomograph, and MR images were subsequently co-registered for each dog brain. The binding capacity for each brain region was assessed by analysis of regional washout rates. Immunohistochemical analysis was performed on brain sections from two dogs using a monoclonal mouse antibody against the amino acid residue 1–16 of Aβ (6E10).

The study population consisted of a group of dogs with a clinical diagnosis of CCD (*n* = 16) and a control group of cognitively normal dogs (*n* = 4). Dogs were recruited from the Small Animal University Hospital at The Department of Clinical Veterinary and Animal Sciences, University of Copenhagen or through referring veterinarians.

The study was approved by the ethics committee at The Department of Clinical Veterinary and Animal Sciences, Faculty of Health and Medical Sciences, University of Copenhagen, Denmark.

### CCD group

The study population consisted of 16 geriatric dogs with a diagnosis of CCD, including nine males and seven females, with an average age of 12.6 years (range 9–17 years; SD 2.2 years). All dogs had a clinical and neurological examination, standard hematological and chemical profiles including thyroid panel showing no evidence of systemic or neurological disease, which could mimic clinical signs of CCD. All dogs had magnetic resonance imaging (MRI) that showed no signs of intracranial lesions that might give rise to clinical signs mimicking CCD. A clinical diagnosis of CCD was established after evaluation with a validated owner questionnaire that targeted signs of CCD and was further supported by the finding of significant brain atrophy on MRI images by means of stereology [Cavalieri principle; (Kiatipattanasakul et al., 1996; Rofina et al., 2006)].

### Control group

The group of control dogs consisted of four dogs aged 5–12 years (mean 8.8 years, SD 3.3), three males and one female. The dogs had a normal clinical and neurological examination and standard hematological and chemical profiles including thyroid panel and showed no signs of systemic disease. All dogs had a normal MRI. The dogs were defined as having normal cognitive behavior based on absence of signs of CCD, as assessed by owner interviews, using the same validated questionnaire used for the CCD group.

Details of the study group are listed in Table 1.

**Table 1:** **Information regarding age, sex, weight, and breed for the study population**.

	Age	Sex	Weight (kg)	Breed
CCD group	13	M	15.6	Beagle
	13	M	2.7	Chihuahua
	12	F	10.0	Cocker Spaniel
	13	F	19.0	Tervueren
	10	F	33.0	German Shepherd
	11	M	12.2	Cairn Terrier
	15	M	8.1	Dachshund
	11	M	15.6	Catalonian sheepdog
	17	M	12.5	Small mix
	14	F	7.9	Jack Russell
	9	M	28.0	Labrador
	11	M	38.0	German Wirehaired Pointer
	13	F	28.0	Labrador
	13	F	22.0	Border Collie
	11	M	20.0	Beagle
	16	M	10.0	Kooikerhondje
Control group	7	M	30.0	Tervueren
	11	M	31.0	Groenendal
	5	M	26.5	Tervueren
	12	F	21.0	Tervueren

### Imaging procedures

PET images of the dog brains were obtained with the HRRT CPS Innovations tomograph mainly used for animal model research. Before PET, each dog was sedated with an intramuscular injection of diazepam 0.4–0.7 mg/kg and methadone 0.2–0.4 mg/kg. Two catheters were placed either in the cephalic or saphenous veins, depending on accessibility, one for the combined administration of anesthesia and maintenance fluids and one for the administration of [^11^C]PiB. Anesthesia was induced by intravenous injection of propofol 4 mg/kg. Following intubation, anesthesia was maintained by a continuous rate infusion of propofol 4–6 mg/kg/h (Grasby 3400 Anesthesia Pump, Groendorf Medicine, Hørsholm, Denmark) and the dog was placed in dorsal recumbency with the head positioned in the scanner field. Each dog was carefully positioned in a deflatable Kroeyer bag in order to minimize individual positioning variability between scans. Respiration was maintained with a ventilator (Hallowell EMC Model 200 Veterinary Anesthesia Ventilator, Hallowell EMC, Pittsfield, MA, USA), and heart rate was monitored by ECG, Oxygen saturation through a pulse oximeter and blood pressure through a pediatric blood pressure cuff placed on one of the hind extremities. One monitor (MP-9000 Mindray Patient Monitor, Shenzhen Mindray Bio-medical Electronics Co., Hamburg, Germany) registered all parameters. The dogs received Lactated Ringer’s solution from an infusion pump (Baxter) throughout the anesthesia at a maintenance rate of 10 ml/kg/h. All dogs were fasted for at least 12 h prior to imaging to reduce anesthetic risk.

Each dog was injected intravenously with 84–333 MBq [*N*-methyl-^11^C]2-(4′-methylaminophenyl)-6-hydroxybenzothiazole ([^11^C]PiB) dissolved in 10 ml of sterile isotonic saline. PET emission data were collected for 90 min preceded by a transmission scan, commencing with the injection of [^11^C]PiB.

We used 30 min MRI with 3 T GE Signa HDxt – Twin speed gradient system to obtain anatomical regions of interest (RIO) (General Electric Medical Systems, Milwaukee, WI, USA). The dogs were placed headfirst into the magnet bore, and an 8-channel GE head coil was secured over the head of the animal. The scanner allowed for acquisition of high-resolution anatomy imaging in contiguous 1 mm thick transverse slices using three dimensional spoiled gradient echo (SPGR), pulse sequence (TE = 2.9 ms, TR = 6.7ms, flip angle = 14°).

### Generation of an MR template brain

No template is available for the co-registration of dogs to a standard 3-D imaging volume, so we constructed a reference template from the MRI anatomical image of one control dog brain. For orientation purposes, the T1-weighted MRI volume of the brain of this dog was linearly registered to an in-house template space of Danish Landrace pigs. The registration was performed using the MINC registration software package from Montreal Neurological Institute (MNI) (Collins et al., 1994). This pig atlas was constructed as described in previous investigations (Maes et al., 1997; Watanabe et al., 2001; Andersen et al., 2005). This control dog brain image was used as a reference space for the subsequent intersubject co-registration of all dog brains in the study. For each dog, the T1-weighted MRI brain volume was manually cropped to initially remove extracerebral tissue, and the image was corrected for non-uniformity in the image intensity (Sled et al., 1998). The MR-image was then registered to the dog model space using a 12 parameter affined transformation (Maes et al., 1997). The images were resampled to isotropic resolution of 0.5 mm 3-D resolution, the cerebral tissue was skull-stripped using the registration, and a brain mask defined on the dog template. We used this skull-stripped MRI as a target for the subsequent MRI-PET registrations of dog brains.

### Reconstruction of [^11^C]PiB images

For each PET image, the dynamic files were averaged over the time dimension and the average image was blurred using a Gaussian kernel to a full width at half maximum (FWHM) of 4.0 mm. The blurred image was then linearly registered using a rigid body affined transformation and mutual information criteria (Collins et al., 1994; Maes et al., 1997). Each dynamic and averaged PET image was spatially normalized using the corresponding concatenated transform from PET to MRI to model space. This spatial normalization was performed using the MNI software package. As no consensus exists on the specific anatomic localization of the different canine brain cortical regions, we used a segregation of cortical regions that largely agrees with the canine stereotaxic brain anatomy atlas (Dua-Sharma et al., 1970).

### Identification of brain regions

**Figure 1:**
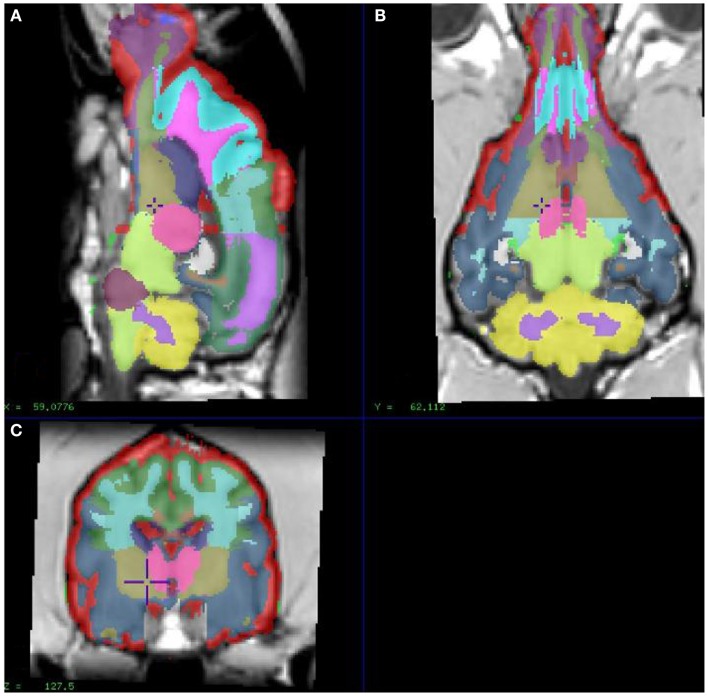
**Showing different sections of a dog brain with the ROI as color coded areas outlined by the authors based on a stereotaxic atlas by Dua-Sharma, in order to extract PET image values for relevant regional brain areas (Dua-Sharma et al., 1970)**. **(A)** Sagittal mid section of a dog brain with identification of ROI. **(B)** Dorsal section of dog brain at the level of the third ventricle and caudate nucleus with identification of ROI. **(C)** Transverse section of dog brain at the level of the third and lateral ventricles and the habenular nucleus with identification of ROI. Color code identifies the gray matter for each ROI used. ROI, regions of interest.

In order to extract image values for relevant regional brain areas, we manually segmented the model dog brain image into specific RIO, which included cortical regions and the cerebellum; frontal, temporal, parietal, and occipital cortices and the cerebellum (Figure 1), with initial tissue segmentation of gray matter, white matter, and CSF classes with an automated method (Cocosco et al., 2003). We then manually parcellated each image into the relevant regions for each tissue class and obtained regional parametric estimates of PiB binding from each of these template regions.

### Parametric mapping of PiB retention

The brain uptake of [^11^C]PiB largely is flow-limited, with an extraction fraction of more than 50% (Blomquist et al., 2008). Therefore, we determined the binding capacity by analysis of regional washout rates, which decline in the presence of binding (Moller et al., 2009). We used washout analysis because unmetabolized [^11^C]PiB disappears rapidly from the circulation and the consequent brief exchange with brain tissue implies that retention of intact tracer in brain tissue depends on the washout rate and not on a continued exchange between compartments of tracer in the tissue and circulation after the period of initial entry of tracer into the tissue, which ends approximately at 4 min after injection (Rodell et al., 2013). Also, we chose this method because we initially had no evidence of the presence of a specific reference region devoid of displaceable binding of [^11^C]PiB in dog brain.

To parametrically map neuroreceptors in relevant cases, a reference region eliminates the need to determine the arterial concentrations of the tracer, particularly when tracer retention is subject washout limitation only (Edison et al., 2012). In brain, amyloid deposits are present in the cerebellum in genetic AD, as cerebral amyloid angiopathy, but also in prion diseases (Villemagne et al., 2012). The choice of an alternative method of identification of a reference region for the analysis of [^11^C]PiB binding therefore was necessary in the present case (Moller et al., 2009; Edison et al., 2012). The chosen method specifically applies to tracers with the kinetic behavior of PiB. It identifies a suitable reference region in the brain when one exists, by locating the area of least [^11^C]PiB binding, considered most suitable for this purpose.

Using the in-house developed software, we extracted mean [^11^C]PiB binding values within specific ROIs from the parametric images and submitted region-based analysis. Figure 1 shows the ROIs used to extract the binding values. The binding potential BP_ND_ of [^11^C]PiB was estimated from the washout measure, in relation to the identified region of reference (Moller et al., 2009). We chose the reference region by first locating the voxels of maximum washout rates, identified by means of the washout index Θ that equals the ratio of the twice to once integrated time-radioactivity records of tracer in the voxels. The faster the washout, and hence the lower the specific binding, the higher the magnitude of the washout index Θ (Moller et al., 2009).

### Histology and immunohistochemistry

Two CCD dog brains underwent *ex vivo* histopathological investigation. After sacrifice, we quickly removed and immersed the brains in 10% neutral buffered formalin until further processing. From each brain, we embedded one hemisphere in alginate and sectioned it coronally into 9 mm thick slabs as directed by the previously obtained MRI/PET images (Sorensen et al., 2000; Bjarkam et al., 2001). We immersed the brain slabs in 30% sucrose for 5 days, froze them with gaseous CO mounted in a cryostat, and then sectioned them further into 20 series of 40 μm thick coronal sections. The resulting sections were mounted on microslides and subsequently stained with Nissl-, AMG, HE, or Congo Red/Thioflavin S, according to standard protocols, or stored freely floating in De Olmos solution for subsequent immunohistochemical staining procedures (Nielsen et al., 2007). We performed immunohistochemical analysis on the freely floating sections with a monoclonal mouse antibody against the amino acid residue 1–16 of Aβ (6E10) (Cat # SIG-39320) (Covance, CA, USA), which does not distinguish between AβPP and Aβ (Aho et al., 2010). We pretreated sections for 10 min with 70% formic acid to expose antigenic sites and then incubated the sections in primary antibody diluted 1:2000 in Tris buffered saline with 1% Triton +0.2% milk overnight at 4°C, before visualization with a secondary goat anti-mouse IgG diluted 1:400 in Tris buffered saline with 1% Triton +0.2% milk for 1 h at room temperature. We visually assessed the staining by light microscopy of 6E10-immunostained sections from the cerebral cortex and the cerebellum.

### Statistical analysis

We analyzed differences among ROI estimates of [^11^C]PiB binding in both gray and white matter with paired-to-sample *t*-test. Group differences were tested with permutation tests, due to the small sample size in the control group, in whole brain, as well as in specific cortical regions. Due to the age span of the cohort, we also assessed group differences after correction for age with regression analysis. A probability of *p* < 0.05 was considered a statistically significant non-random result.

## Results

**Figure 2:**
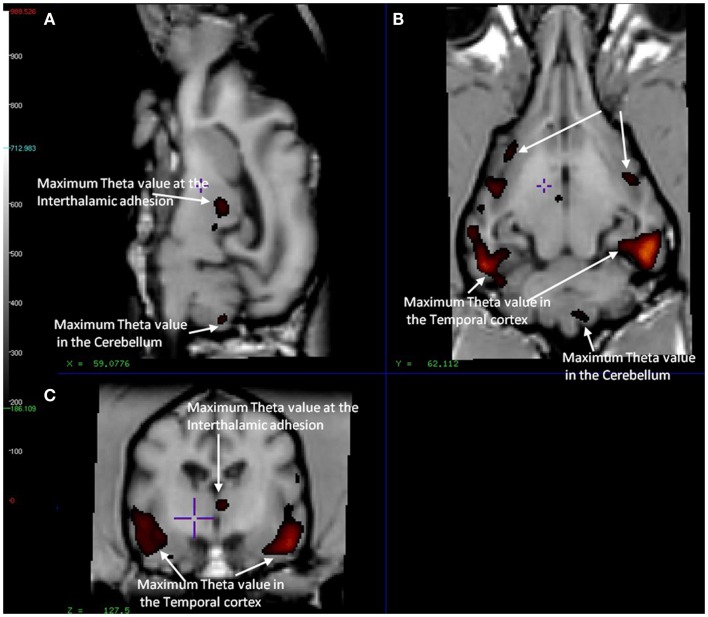
**Magnetic resonance imaging of the brain of CCD dogs with reference region of max average theta value (red/dark red) identical with an area of maximal washout characteristics of [11C]PiB highlighted in red and therefore minimum binding of [11C]PiB**. **(A)** Mid-sagittal section of dog brain showing an area of max theta value (red/dark red) and therefore minimum [11C]PiB binding in the thalamic adhesion and the cerebellum. **(B)** Dorsal section of dog brain at the level of the third ventricle and caudate nucleus. Showing areas of max theta value (red/dark red) in the parietal and temporal lobes. **(C)** Transverse section of dog brain at the level of the third and lateral ventricles and the habenular nuclei. Identifying areas of maximum theta (red/dark red) and therefore minimum [11C]PiB binding in the temporal lobes and the thalamic adhesion.

In the CCD group [^11^C]PiB binding was significantly higher in the gray matter of the cerebellum, than in the frontal (*p* < 0.001), temporal (*p* < 0.001), parietal (*p* < 0.001), and occipital (*p* < 0.001) lobes. Thus, the voxel maps of the washout index (Θ(*T*)) values did not confirm the prediction of the cerebellum as a suitable reference region. We chose the reference area to be all voxels in which the value of Θ equaled or exceeded 95% of the maximum value of Θ (2170 s). Surprisingly, we found this reference region to be situated in the temporal cortex (Figure 2). Greater PiB binding was seen in the gray matter of the occipital lobe, compared with the frontal and temporal lobes, with *p* = 0.03 and *p* = 0.003, respectively, for these comparisons. In addition, the [^11^C]PiB reached significantly higher binding in the gray matter in the parietal lobe than in the temporal lobe (*p* < 0.001) (Table 2). The maps revealed differential regional [^11^C]PiB binding, with the highest binding in the cerebellum.

**Table 2:** **Mean BP_ND_ for [^11^C]PiB in different ROI determined by the Hypotime method for gray and white matter in the CCD group**.

	Cerebellum	Frontal cortex	Temporal cortex	Parietal cortex	Occipital cortex
Gray matter (mean BP_ND)_	0.35	0.23	0.22	0.24	0.26
Cerebellum vs. other ROI (*p* <)		0.001	0.001	0.001	0.001
White matter (mean BP_ND)_	0.39	0.23	0.25	0.24	0.25
Cerebellum vs. other ROI (*p* <)		0.001	0.001	0.001	0.001

*BP_ND_, binding potential; PiB, Pittsburg compound B; CCD, canine cognitive dysfunction; ROI, regions of interest*.

**p*-Values for difference in mean BP_ND_ between different ROI*.

For white matter, significantly higher [^11^C]PiB binding occurred in the cerebellum compared to the frontal, temporal, parietal, and occipital lobes (*p* < 0.001) (Table 2), with mean estimates of [^11^C]PiB binding potentials (BP_ND_) listed in Table 3 for all ROIs.

**Table 3:** **showing mean BP_ND_ for [^11^C]PiB determined by the Hypotime method**.

ROI Dog id	Cerebellum Gray matter Mean BP_ND_	Cerebellum White matter Mean BP_ND_	Frontal lobe Gray matter Mean BP_ND_	Frontal lobe White matter Mean BP_ND_	Temporal lobe Gray matter Mean BP_ND_	Temporal lobe White matter Mean BP_ND_	Parietal lobe Gray matter Mean BP_ND_	Parietal lobe White matter Mean BP_ND_	Occipital lobe Gray matter Mean BP_ND_	Occipital lobe White matter Mean BP_ND_
**CCD GROUP**										
1108	0.36918	0.41686	0.25736	0.27780	0.23450	0.27309	0.28551	0.29090	0.32647	0.32177
3252	0.29433	0.33419	0.22862	0.22355	0.21422	0.25950	0.24485	0.22384	0.21768	0.22302
18361	0.43665	0.34678	0.39350	0.37690	0.41874	0.38073	0.39839	0.34483	0.32867	0.20188
24503	0.36242	0.37585	0.30868	0.33663	0.39035	0.39897	0.36618	0.38278	0.33793	0.39035
27168	0.38462	0.51318	0.19780	0.20738	0.16561	0.21138	0.19474	0.20348	0.20727	0.22843
27055	0.28487	0.29230	0.24726	0.24043	0.18862	0.19913	0.22122	0.19580	0.24063	0.22283
27777	0.37050	0.50813	0.16553	0.15198	0.16889	0.17265	0.16966	0.16724	0.21399	0.21677
26494	0.30800	0.28431	0.19754	0.16082	0.15024	0.16348	0.17263	0.16019	0.17932	0.16612
26281	0.16585	0.17314	0.15036	0.11858	0.15762	0.16479	0.20272	0.19863	0.20245	0.24843
27123	0.52081	0.55678	0.37785	0.38499	0.46538	0.55397	0.53145	0.54140	0.46887	0.48173
29415	0.32971	0.42708	0.19442	0.20514	0.14215	0.15619	0.17425	0.17719	0.17228	0.15003
24324	0.49174	0.71424	0.29807	0.30279	0.31701	0.40795	0.33796	0.34689	0.41262	0.34943
23620	0.28615	0.25388	0.16042	0.15720	0.13067	0.14567	0.14248	0.12801	0.22083	0.19170
26410	0.32704	0.41380	0.13108	0.12207	0.11948	0.15642	0.11477	0.09918	0.19422	0.15573
20041	0.28830	0.26793	0.18012	0.15675	0.13928	0.15471	0.15767	0.14654	0.16238	0.15333
29165	0.36743	0.40270	0.20472	0.21982	0.18953	0.23377	0.20293	0.21934	0.26152	0.24769
**CONTROL GROUP**										
31036	0.25143	0.31536	0.17511	0.18550	0.12698	0.15761	0.17070	0.18368	0.18761	0.19417
31097	0.32839	0.48010	0.27697	0.26922	0.21317	0.29735	0.26637	0.28353	0.29711	0.28859
31140	0.36561	0.37084	0.26716	0.30053	0.19079	0.23095	0.27198	0.26645	0.26197	0.26120
27351	0.26679	0.28976	0.19716	0.18982	0.14679	0.17281	0.17080	0.15745	0.20649	0.18540

*BP_ND_. Mean binding potential of [^11^C]PiB calculated using the Hypotime method*.

**Figure 3:**
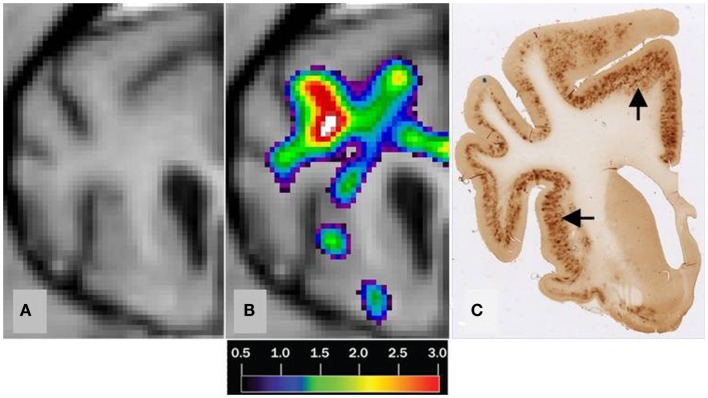
**Correlation of MRI [^11^C]PiB PET imaging and histopathology of a transverse brain section at the level of the rostral commissure and the corpus striatum**. The brain is from a 17-year old small mixed breed male dog with clinical diagnosis of CCD. **(A)** Transverse MRI of brain from CCD dog at the level of rostral commissure and the corpus striatum **(B)** MRI from picture 1 and corresponding [^11^C]PiB PET imaging. [^11^C]PiB binding is indicated by a color scale with red indicating high retention and black indicating low retention. Calculated using the Hypotime method. **(C)** Corresponding section to 1 and 2 immunostained with amyloid (Aβ) monoclonal antibody 6E10. Dark areas identifies positive areas of immunostaining using 6E10 indicating and thereby either Aβ and AβPP deposits or both (Marked with arrows). MRI, magnetic resonance imaging; PiB, Pittsburgh compound B; PET, positron emission tomography; CCD, canine cognitive dysfunction.

**Figure 4:**
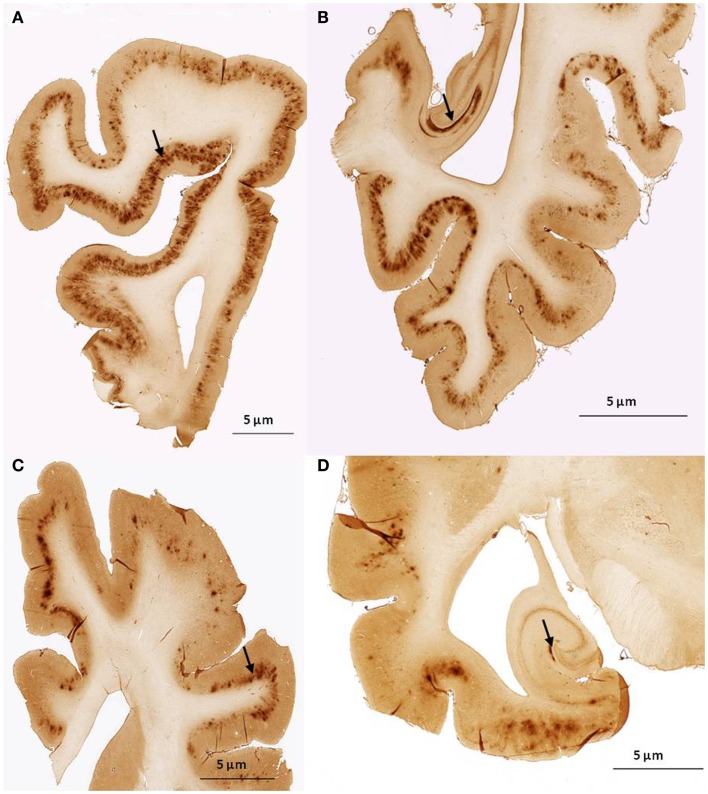
**Histological sagittal sections of the frontal and temporal lobes including the hippocampus**. Sections are immunostained with amyloid (Aβ) monoclonal antibody 6E10 **(A,B)** are histological section from the frontal and temporal lobe, respectively. Obtained from a 17-year old small mixed breed with signs of cognitive dysfunction. The sections exhibit diffuse staining of cortical gray matter in the frontal and temporal cortex and marked staining of the perforant pathway in the hippocampus. **(C,D)** are histological section from the frontal and temporal lobe, respectively. Obtained from a 13-year old collie with signs of cognitive dysfunction. The sections exhibit diffuse staining of the cortical gray matter in the frontal and temporal cortex. The perforant pathway in the hippocampus only demonstrate marked staining in a very small area compared to **(B)**.

**Figure 5:**
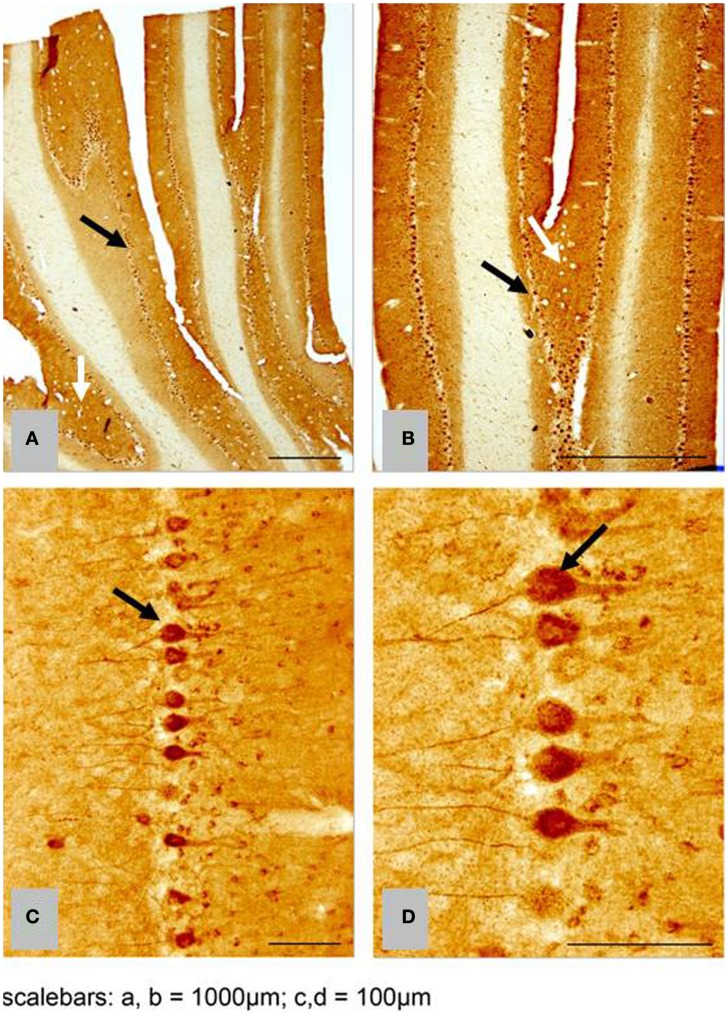
**Histological horizontal sagittal sections through the anterior lobe of the cerebellum**. Obtained from a 13-year old female Border Collie with clinical signs of canine cognitive dysfunction. **(A,B)** Low magnification view immunostained with amyloid (Aβ) monoclonal antibody 6E10 showing the presence of marked immunostaining of Purkinje cells (black arrows), but also exhibiting diffuse staining of the granular and molecular layer (white arrows). **(C,D)** High magnification view of Purkinje cells in **(A,B)** showing intracellular accumulation of Aβ positive material. CCD, canine cognitive dysfunction.

We found no significant differences of [^11^C]PiB binding between the CCD and control groups in any ROI. Neither did we find any correlation between the *in vivo* [^11^C]PiB accumulation in images obtained with PET, and the *ex vivo* matched measures of Aβ and AβPP staining post-mortem (Figure 3), consistent with the observation that neocortex had very little signal from [^11^C]PiB, in contrast to the extensive immunohistochemical staining of diffuse extracellular Aβ and AβPP deposits (Figure 4). The one region of the brain that did have significant labeling with [^11^C]PiB, the cerebellum, also had marked intracellular staining of Aβ or AβPP, or both, in the Purkinje cells, and diffuse Aβ and AβPP staining of the granule cell and molecular layers (Figure 5). The two dogs with immunohistochemical staining did not show any Nissl, AMG, HE, or Congo Red/Thioflavin positive amyloid deposits.

## Discussion

We present the first evidence that PET images of the distribution of [^11^C]PiB retention in brain of dogs with CCD fundamentally are different from the images obtained in most humans with AD. Unlike the general evidence of uptake in brain of humans with AD, the evidence in this study revealed the highest uptake in the cerebellum of the dogs with CCD and not in the neocortical regions. We noted the least retention of the biomarker in the temporal cortex (Figure 2). To obtain this finding, we mapped unto the brain images an index of the biomarker washout, the washout index Θ, which rises with the rate of washout. From the retention of the biomarker, we concluded that the cerebellum gray matter in the dog brain would not be an appropriate reference region for binding of [^11^C]PiB in dogs, a finding that is at variance with studies of human AD, in whom the cerebellum shows little [^11^C]PiB binding and is an appropriate reference region (Klunk et al., 2004; Ikonomovic et al., 2008; Rodell et al., 2013).

Comparison with the images of the histological material revealed no correlation between the images of [^11^C]PiB and the Aβ and AβPP deposits in post-mortem brain, despite marked intracellular staining of Aβ/AβPP in the Purkinje cells and diffuse staining of the granule and molecular cell layers. The marked intracellular staining implies intracellular involvement of amyloid processing in the dog brain (Figure 5).

We used the washout-dependent time variable Θ to identify an alternative region of reference and to calculate binding potentials. The key to the analysis is the time variable Θ, which is related to and serves as an index of the rate of washout and hence of the degree of expansion of the apparent volume of distribution of the tracer occasioned by binding. By this approach, we found areas of rapid clearance in small areas of the temporal cortex (Figure 2), which we chose as regions of reference. As defined by us, the borders of the reference region enclosed an area in which the values of Θ equaled or exceeded 95% of the maximum mean value of the time variable Θ of 2170 s. This reference was used throughout the subsequent analysis to determine the binding potentials relative to non-displaceable accumulation (BP_ND_)_._

We did note diffuse Aβ/AβPP immunostaining of both frontal and temporal cortices with 6E10, however these deposits did not bind [^11^C]PiB to the same degree as the deposits in the cerebellum, implying that dogs with CCD are not afflicted with a typical form of AD (Figure 4). Because the major part of amyloid is not usually found in cerebellum of humans with AD, unlike in the brains of the present group of CCD dogs, CCD may instead be a better model of genetic AD in humans, or hereditary cerebral hemorrhage with amyloidosis and prion diseases, also in humans (Watanabe and Duchen, 1993; Ghiso and Frangione, 2001; Klunk et al., 2007). However, these hypotheses are speculative and yet to be confirmed in families of dogs.

The high degree of [^11^C]PiB binding in the cerebellum, with few if any extracellular deposits and large amounts of intracellular Aβ or AβPP, or both, suggests that [^11^C]PiB binding may be used to distinguish between intracellular Aβ and AβPP deposits in the dog cerebellum. Aged dogs with CCD spontaneously exhibit diffuse Aβ deposits in the cerebral cortex that are similar to those found in early AD brains (Cummings et al., 1993, 1996; Satou et al., 1997; Anderson et al., 2000; Head et al., 2008; Yu et al., 2011). In addition, the diffuse Aβ deposits in the canine brain are immunopositive for Aβ_1–42_ and do not stain with Congo red and thioflavine, unlike proven cerebral amyloid angiopathy (Uchida et al., 1992; Cummings et al., 1993, 1996; Satou et al., 1997).

The high proportion of diffuse to compact Aβ deposits in dogs with CCD theoretically should not pose a problem, because previous investigations show that [^11^C]PiB in tissue from AD patients also recognizes diffuse Aβ deposits in addition to compact Aβ deposits (Klunk et al., 2003; Lockhart et al., 2007). Hence [^11^C]PiB in principle would be a tracer suitable for outlining the distribution of Aβ deposits in the aged dog with CCD with a cranial to caudal distribution. The results point to a pattern of [^11^C]PiB retention in the dog brain, which is occasionally seen in humans. We also found a discrepancy between the distributions of Aβ and AβPP, when we visually correlated [^11^C]PiB images with regionally matched images of the post-mortem histological material stained with 6E10. In a study of a cohort of 10 AD patients, one patient who met the histopathological criteria for AD had PiB-refractory PET images (Rosen et al., 2010). This patient had more vascular Aβ, higher levels of insoluble Aβ_1–40_ and Aβ_1–42_, and a higher ratio of Aβ_1–40_ to Aβ_1–42_, compared to brain tissue from the nine other AD patients.

Studies of [^11^C]PiB imaging in PS1/APP mice and post mortem histological analysis have shown diverging results of different attempts to find a correlation between the [^11^C]PiB images and Aβ deposits *in vitro* (Klunk et al., 2005a; Manook et al., 2012). The negative results may be explained in part by the presence of lower affinity PiB binding sites on Aβ deposits in the transgenic mouse brains than in AD brains (Klunk et al., 2005a; Toyama et al., 2005). This explanation implies that a part of the PiB binding in mouse and dog brains may be selective for a specific conformation of Aβ deposits found in AD patients at the low nanomolar concentrations used in PET studies. This hypothetical selectivity of PiB is consistent with another study of transgenic mice, in which it was concluded that the detectability of amyloid by PiB depends on the accumulation of specific Aβ subtypes. This explanation was proposed as well in a study of primate brain homogenates, which showed markedly reduced binding to PiB compared to human brain homogenates (Rosen et al., 2011).

This study gave evidence that the distribution of [^11^C]PiB retention in dogs with CCD fundamentally is different from that seen in humans with AD. The results of the study support previous studies showing that [^11^C]PiB is a complex tracer in animals and humans and more investigations of this radiotracer, including displacement studies are needed.

## Conflict of Interest Statement

The research in this paper was conducted in the absence of any commercial or financial relationships that could be construed as a potential conflict of interest to authors.
